# Increased immunization coverage addresses the equity gap in Nepal

**DOI:** 10.2471/BLT.16.178327

**Published:** 2017-02-02

**Authors:** Ashish KC, Viktoria Nelin, Hendrikus Raaijmakers, Hyung Joon Kim, Chahana Singh, Mats Målqvist

**Affiliations:** a United Nations Children’s Fund, Nepal Country Office, PO Box 1187, UN House, Pulchowk, Lalitpur, Kathmandu, Nepal.; b Department of Women’s and Children’s Health, Uppsala University, Uppsala, Sweden.

## Abstract

**Objective:**

To compare immunization coverage and equity distribution of coverage between 2001 and 2014 in Nepal.

**Methods:**

We used data from the Demographic and Health Surveys carried out in 2001, 2006 and 2011 together with data from the 2014 Multiple Indicator Cluster Survey. We calculated the proportion, in mean percentage, of children who had received bacille Calmette–Guérin (BCG) vaccine, three doses of polio vaccine, three doses of diphtheria–pertussis–tetanus (DPT) vaccine and measles vaccine. To measure inequities between wealth quintiles, we calculated the slope index of inequality (SII) and relative index of inequality (RII) for all surveys.

**Findings:**

From 2001 to 2014, the proportion of children who received all vaccines at the age of 12 months increased from 68.8% (95% confidence interval, CI: 67.5–70.1) to 82.4% (95% CI: 80.7–84.0). While coverage of BCG, DPT and measles immunization statistically increased during the study period, the proportion of children who received the third dose of polio vaccine decreased from 93.3% (95% CI: 92.7–93.9) to 88.1% (95% CI: 86.8–89.3). The poorest wealth quintile showed the greatest improvement in immunization coverage, from 58% to 77.9%, while the wealthiest quintile only improved from 84.8% to 86.0%. The SII for children who received all vaccines improved from 0.070 (95% CI: 0.061–0.078) to 0.026 (95% CI: 0.013–0.039) and RII improved from 1.13 to 1.03.

**Conclusion:**

The improvement in immunization coverage between 2001 and 2014 in Nepal can mainly be attributed to the interventions targeting the disadvantaged populations.

## Résumé

**Objectif:**

Comparer la couverture vaccinale et la répartition équitable de la couverture entre 2001 et 2014 au Népal.

**Méthodes:**

Nous avons utilisé des données provenant d'enquêtes démographiques et sanitaires réalisées en 2001, 2006 et 2011, ainsi que des données tirées d'une enquête par grappes à indicateurs multiples menée en 2014. Nous avons calculé la proportion, en pourcentage moyen, d'enfants ayant reçu le vaccin bilié de Calmette et Guérin (BCG), les trois doses du vaccin antipoliomyélitique, les trois doses du vaccin contre la diphtérie, la coqueluche et le tétanos (DCT) et le vaccin contre la rougeole. Afin de mesurer les inégalités entre les différents quintiles de richesse, nous avons calculé l'indice de pente d'inégalité (SII) et l'indice relatif d'inégalité (RII) dans le cadre de toutes les enquêtes.

**Résultats:**

De 2001 à 2014, la proportion d'enfants ayant reçu tous les vaccins à l'âge de 12 mois est passée de 68,8% (intervalle de confiance, IC, à 95%: 67,5–70,1) à 82,4% (IC à 95%: 80,7–84,0). Si la couverture des vaccins BCG, DCT et contre la rougeole a statistiquement augmenté au cours de la période étudiée, la proportion d'enfants ayant reçu la troisième dose du vaccin antipoliomyélitique a diminué de 93,3% (IC à 95%: 92,7–93,9) à 88,1% (IC à 95%: 86,8–89,3). L'amélioration la plus importante en matière de couverture vaccinale a été observée au sein du quintile de richesse le plus bas (de 58% à 77,9%), le quintile le plus riche connaissant seulement une amélioration de 84,8% à 86,0%. Dans le cas des enfants ayant reçu tous les vaccins, le SII est passé de 0,070 (IC à 95%: 0,061–0,078) à 0,026 (IC à 95%: 0,013–0,039) et le RII de 1,13 à 1,03.

**Conclusion:**

L'amélioration de la couverture vaccinale entre 2001 et 2014 au Népal résulte essentiellement des interventions ciblant les populations défavorisées

## Resumen

**Objetivo:**

Comparar la cobertura de inmunización y la distribución de la equidad de cobertura entre 2001 y 2014 en Nepal.

**Métodos:**

Se utilizaron datos de las Encuestas de Demografía y Salud realizadas en 2001, 2006 y 2011, junto con datos de la Encuesta de Conglomerados de Indicadores Múltiples de 2014. Se calculó la proporción (en porcentaje medio) de niños que habían recibido la vacuna del bacilo de Calmette-Guérin (BCG), tres dosis de la vacuna antipoliomielítica, tres dosis de la vacuna contra la difteria, la tos ferina y el tétanos (DPT) y la vacuna contra el sarampión. Para medir la falta de equidad entre los quintiles de riqueza, se calcularon el índice de desigualdad de la pendiente (IDP) y el índice relativo de desigualdad (IRD) en todas las encuestas.

**Resultados:**

De 2001 a 2014, el porcentaje de niños que recibieron todas las vacunas a la edad de 12 meses aumentó de un 68,8% (intervalo de confianza, IC, del 95%: 67,5–70,1) a un 82,4% (IC del 95%: 80,7–84,0). Estadísticamente, si bien la cobertura de inmunización contra BGC, DPT y sarampión aumentó durante el periodo de estudio, el porcentaje de niños que recibieron la tercera dosis de la vacuna antipoliomielítica descendió de un 93,3% (IC del 95%: 92,7–93,9) a un 88,1% (IC del 95%: 86,8–89,3). El quintil de riqueza más pobre fue el que mostró la mayor mejora en la cobertura de inmunización, de un 58% a un 77,9%, mientras que el más rico mejoró únicamente de un 84,8% a un 86,0%. El IDP para los niños que recibieron todas las vacunas aumentó de un 0,070 (IC del 95%: 0,061-0,078) a un 0,026 (IC del 95%: 0,013–0,039) y el IRD aumentó de un 1,13 a un 1,03.

**Conclusión:**

La mejora de la cobertura de inmunización entre 2001 y 2014 en Nepal puede atribuirse principalmente a las intervenciones orientadas a los sectores demográficos desfavorecidos.

## ملخص

**الغرض:**

مقارنة تغطية التمنيع وتوزيع الإنصاف في خدمات التغطية في الفترة ما بين عاميّ 2001 و2014 في نيبال.

**الطريقة:**

استخدمنا البيانات المستخرجة من المسوح الصحية الديموغرافية التي تمت في سنوات 2001، و2006، و2011 مع البيانات المستخرجة من المسوح العنقودية متعددة المؤشرات. قمنا بحساب النسبة المئوية للأطفال الذين تلقوا لقاح عصيات كالميت غيران (BCG)، وثلاث جرعات من لقاح شلل الأطفال، وثلاث جرعات من لقاح الدفتريا والسعال الديكي والتيتانوس (اللقاح الثلاثي) ولقاح الحصبة. ولحساب حالات التفاوت بين الفئات الخمسية السكانية، قمنا بحساب مؤشر منحدر عدم المساواة (SII) والمؤشر النسبي لعدم المساواة (RII) لجميع المسوح.

**النتائج:**

ارتفعت نسبة الأطفال ممن تلقوا التطعيمات في عمر 12 شهرًا في الفترة من سنة 2001 إلى 2014 من ‏68.8‏% (بنسبة أرجحية يبلغ مقدارها ‏95‏%: 67.5 – 70.1) إلى ‏82.4‏% (بنسبة أرجحية يبلغ مقدارها 95‏‏%: 80.7 – 84.0). بينما ارتفعت نسبة تغطية التمنيع لعصيات كالميت غيران (BCG)، والدفتريا والسعال الديكي والتيتانوس والحصبة إحصائيًا خلال فترة الدراسة، فقد انخفضت نسبة الأطفال ممن تلقوا جرعة تطعيم شلل الأطفال من 93.3‏‏% (بنسبة أرجحية يبلغ مقدارها ‏95‏%: 92.7 – 93.9) إلى 88.1‏‏% (بنسبة أرجحية يبلغ مقدارها 95‏‏%: 86.8 – 89.3). وأظهرت الفئات الخمسية السكانية الفقيرة تحسنًا كبيرًا في تغطية التمنيع من ‏58‏% إلى ‏77.9‏%، بينما تحسنت الفئات الخمسية السكانية الغنية من 84.8‏‏% إلى 86.0‏‏% فقط. وتحسن مؤشر منحدر عدم المساواة للأطفال ممن تلقوا التطعيمات من 0.070 (بنسبة أرجحية يبلغ مقدارها 95‏‏%: 0.061 – 0.078) إلى 0.026 (بنسبة أرجحية يبلغ مقدارها ‏95‏‏%: 0.013 – 0.039) وتحسن المؤشر النسبي لعدم المساواة من 1.13 إلى 1.03.

**الاستنتاج:**

يمكن إرجاء التحسن في تغطية التمنيع في الفترة من 2001 و2014 إلى التدخلات التي تستهدف القطاعات السكانية المحرومة.

## 摘要

**目的:**

旨在比较 2001 年至 2014 年间尼泊尔的免疫覆盖率和覆盖率分布平等性。

**方法:**

我们采用 2001 年、2006 年和 2011 年开展的人口健康调查的数据，以及 2014 年多指标群体调查的数据。 我们计算了接受卡介苗 (BCG)、三剂脊髓灰质炎疫苗、三剂百白破 (DPT) 疫苗和麻疹疫苗的儿童比例（即平均百分比）。 为了衡量财富五分位人群之间的不平等性，我们计算了所有调查的不平等斜率指数 (SII) 和不平等相对指数 (RII)。

**结果:**

从 2001 年至 2014 年，在 12 个月大时接受全部疫苗的儿童比例从 68.8%（95% 置信区间，CI： 67.5–70.1）提高至 82.4%（95% CI： 80.7–84.0)。 虽然卡介苗 (BCG)、百白破 (DPT) 和麻疹的免疫统计数据在研究期间有所提高，但接受第三剂脊髓灰质炎疫苗的儿童比例从 93.3% (95% CI: 92.7–93.9) 降低至 88.1% (95% CI: 86.8–89.3)。 财富五分位中最贫穷人口的免疫覆盖率的提升最为显著，即，从 58% 至 77.9%。而财富五分位中最富有人口仅由 84.8% 提升至 86.0%。 接受全部疫苗的儿童的不平等斜率指数 (SII) 从 0.070 (95% CI: 0.061–0.078) 提高至 0.026 (95% CI: 0.013–0.039)，不平等相对指数 (RII) 从 1.13 提高至 1.03。

**结论:**

2001 年至 2014 年尼泊尔免疫覆盖率的提高主要归功于针对弱势群体的干预措施。

## Резюме

**Цель:**

Провести сравнение уровня охвата иммунизацией и равномерности распределения охвата в период между 2001 и 2014 годами в Непале.

**Методы:**

Авторы использовали данные демографических исследований в области здравоохранения, проведенных в 2001, 2006 и 2011 годах, вместе с данными кластерного обследования 2014 года со множественными показателями. Авторы рассчитали пропорцию в среднем процентном значении детей, которым была введена вакцина против туберкулеза (бацилла Кальметта-Герена (БЦЖ)), три дозы вакцины против полиомиелита, три дозы вакцины против дифтерии, коклюша, столбняка (АКДС) и вакцина против кори. Чтобы измерить уровень неравенства между квинтилями дохода, для всех обследований был рассчитан индекс наклона неравенства (slope index of inequality, SII) и относительный индекс неравенства (relative index of inequality, RII).

**Результаты:**

С 2001 по 2014 год доля детей, получивших все вакцины в возрасте 12 месяцев, увеличилась с 68,8% (95%-й доверительный интервал, ДИ: 67,5–70,1) до 82,4% (95%-й ДИ: 80,7–84,0). В то время как охват БЦЖ, АКДС и иммунизацией против кори статистически увеличился в течение периода исследования, доля детей, получивших третью дозу вакцины против полиомиелита, уменьшилась с 93,3% (95%-й ДИ: 92,7–93,9) до 88,1% (95%-й ДИ: 86,8–89,3). В беднейшем квинтиле дохода произошло наибольшее увеличение охвата иммунизацией — с 58 до 77,9%, в то время как в наиболее богатом квинтиле охват увеличился только с 84,8 до 86,0%. Для детей, которые получили все вакцины, индекс SII изменился с 0,070 (95%-й ДИ: 0,061–0,078) до 0,026 (95%-й ДИ: 0,013–0,039), а индекс RII изменился с 1,13 до 1,03.

**Вывод:**

Увеличение охвата иммунизацией в период между 2001 и 2014 годами в Непале в первую очередь можно связать с мероприятиями, ориентированными на малообеспеченные группы населения.

## Introduction

Immunization is a proven, cost-effective intervention to reduce morbidity and mortality from vaccine-preventable diseases.^1^ Each year immunization averts 2.5 million deaths in children younger than 5 years.^2^ Globally in 2011, 103 million (83% of total) children received all three doses of diphtheria–pertussis–tetanus (DPT3) vaccine, but an estimated 22 million children did not complete such vaccination.^1^ Gaps in immunization coverage exist between and within countries, and in some places, the gap is increasing. For example, the average DPT3 coverage in low-income countries was 15 percentage points lower than that of high-income countries in 2011.^1^^,^^3^^,^^4^

To reach universal immunization coverage and to increase equity, countries need to focus on targeted interventions that reach the most disadvantaged populations, rather than only focusing on increasing coverage at the national level.^1^^,^^5^ For example, countries with the most rapid increase in overall coverage of measles vaccination also show the greatest improvement in coverage of the population in the poorest wealth quintile.^6^

One intervention to reach disadvantaged populations is by targeted communication, which aims to create a demand for immunization in a population. Communication through community mobilization, where influential local leaders support vaccination, could have a positive influence on the community’s trust and willingness to vaccinate their children.^7^ Which communication intervention to use in hard-to-reach populations to improve immunization coverage has to be based on which vaccination barriers these populations are facing.

To increase immunization coverage in Nepal, the government has invested in efforts and resources to improve the service delivery system of the national immunization programme.^8^ During the last two decades, national health policies and a health sector strategy have been implemented to improve coverage and the health and survival of children.^9^^–^^11^ One of the main strategic pillars of the *National communication strategy on maternal, newborn, child health 2011–2016*, is community mobilization of unvaccinated and under-vaccinated communities to increase coverage.^12^ Furthermore, to increase access to vaccination in geographic areas with low coverage, the government has identified bottlenecks in supply of services and has mobilized local resources to intensify outreach clinics and mobile clinics.^13^^–^^15^

The Nepalese National Immunization Programme, created in 1979, aims to vaccinate all children in the country. The programme is guided by a comprehensive multi-year plan for 2017–2022.^16^ According to this plan, the public sector is the primary provider of immunization services, although the private sector is increasingly providing such services. The government provides all vaccine included in the programme free-of-charge. In 1989, the programme provided immunization services for six vaccine-preventable diseases, which were oral polio vaccine, DPT vaccine, tetanus toxoid, bacille Calmette–Guérin (BCG) for tuberculosis and measles vaccine. Between 2000 and 2015, the programme introduced six new vaccines: hepatitis B vaccine, *Haemophilus influenza B* vaccine, rubella vaccine, Japanese encephalitis vaccine, injectable polio vaccine and pneumococcal vaccine. In 2015, the programme provided immunization for 11 vaccine-preventable diseases (Table 1).

**Table 1:** National immunization programme, Nepal, 2015

Vaccine	No. of doses	Age at vaccination
BCG	1	At birth
Pentavalent**^a^**	3	6, 10 and 14 weeks
Oral polio**^b^**	3	6, 10 and 14 weeks
Measles rubella	2	9 and 15 months
Injectable polio^b^	1	14 weeks
Pneumococcal	3	6 and 10 weeks and 9 months
Japanese encephalitis	1	12 months
Tetanus toxoid	2	N/A^c^

BCG: bacille Calmette–Guérin; N/A: not applicable.

^a^ Includes diphtheria, pertussis, tetanus, hepatitis B and *Haemophilus influenza* type B vaccines.

^b^ Children receives both oral and injectable polio vaccines.

^c^ Administrated to pregnant women.

Health facilities and outreach clinics are the main providers of immunization services in Nepal. Each district has about 3–5 outreach clinics per village. In urban areas of the district, government health facilities, municipal clinics, private hospitals and private clinics deliver immunization services, in coordination and collaboration with the district health offices.

The objective of this study was to investigate changes in immunization coverage and equity gaps between 2001 and 2014, by using data from nationwide surveys.

## Methods

### Survey data

We used national survey data from Demographic and Health Surveys (DHS) for the years 2001,^17^ 2006^18^and 2011^19^ and the 2014 Multiple Indicator Cluster Survey (MICS).^20^ Standardized sampling techniques were used in the four surveys to cover all areas of Nepal. The DHS and MICS methods apply different sampling frames, but all surveys are considered to be nationally representative, and can thus be considered comparable. Further details about the surveys can be found elsewhere.^17^^–^^20^ The 2014 MICS is the first nationally representative MICS performed in Nepal. DHS data has been accessed with permission from ORC Macro and MICS has been accessed and analysed with permission from the United Nations Children’s Fund. From the surveys, we included data on children younger than 5 years.

### Variables

For comparison reasons, we chose immunizations that were included in all four surveys. The variables comprised of: BCG vaccination at birth; three doses of polio vaccine at 1, 2 and 3 months of age; three doses of DPT vaccine at 1, 2 and 3 months of age; and measles vaccine at 9 months of age. To calculate fully immunized children, we created a composite variable for children who had received all of these vaccines at the age of 12 months.

To measure inequity, driven by socioeconomic determinants,^21^ we used the theoretical framework of the Commission on Social Determinants of Health. This framework emphasizes people’s social position based on structural determinants as the main driver of inequity.^21^ To evaluate levels of inequity in immunization coverage, we stratified the immunization coverage data by socioeconomic determinants, including maternal education, area of residence, sex of child and family wealth. We did not include intermediary determinants, such as migration and maternal employment status. We dichotomized the determinants to detect differences between the most advantaged and the most disadvantaged populations. Maternal education was dichotomized as women with no education and women with a primary education or higher. Family areas of residence were divided between mountain areas and hill or terrain regions; the most disadvantaged families were those living in mountain areas. The families’ wealth index was constructed through principal component analysis of scores based on families’ ownership of durable assets, housing characteristics and access to services. From these indexes, we created wealth quintiles. These quintiles were then ranked from bottom to top, as poorest (first), poorer (second), middle (third), richer (fourth) and richest (fifth). To detect inequity between wealth quintiles, dichotomization was done as follows: the poor belonging to the poorest quintile, and the non-poor belonging to the other four quintiles. Even though the framework of the Commission on Social Determinants of Health considered ethnicity and caste as structural determinants, we were not able to include these variables in the analyses, since they were not captured in MICS.

### Data analysis

For each vaccine we calculated the proportion of children who had received the recommended immunization for their age. For the composite variable, we calculated the proportion of children who had received all vaccination at the age of 12 months.

We calculated the slope index of inequality (SII) and the relative index of inequality (RII), which are two regression-based measures, for immunization coverage based on wealth quintiles. Both indexes take into account the whole socioeconomic distribution and remove variability in the size of socioeconomic groups as a source of variation in the magnitude of inequalities in health. SII is the absolute difference of immunization coverage between the richest and the poorest wealth quintile.^22^ RII is the ratio of the immunization coverage estimate between wealth quintiles, calculated on the basis of the systematic association between coverage estimate and the socioeconomic status for all wealth quintiles.^22^ An RII value of 1 indicates equity, while any value larger than 1 indicates inequity.

To analyse if there was an association between any of the socioeconomic determinants and immunization coverage, we used logistic regression models. We used univariate logistic regression to detect associations between coverage and socioeconomic variables, such as wealth quintile, geographic location, sex of child and maternal education. For those variables where we detected an association in the univariate logistic regression model, we did a multivariate logistic regression to adjust the effect of each socioeconomic variable on the immunization coverage. Results are presented as adjusted odds ratios (aORs). In this analysis, the sex of the child is an effect modifier of immunization coverage. Therefore, we checked whether sex of the child was associated with immunization coverage and found that no multicollinearity was detected during the initial analyses testing for effect modification.

We used SPSS version 20.0 (IBM, Chicago, United States of America) for the statistical analyses and a *P*-value of 0.05 was considered significant.

## Results

After deleting survey responses for children with no information on immunization, we included 6931 (2001 DHS survey), 5783 (2006 DHS survey), 5306 (2011 DHS survey) and 3085 (2014 MICS survey) children in the analyses.

Overall, the surveys showed improvements in immunization coverage between 2001 and 2014 (Table 2), with significant increases in BCG, DPT and measles immunizations. Coverage of polio immunization did however show a declining trend over the same period. Also coverage of the second and third dose of DPT vaccine decreased in the latest survey in 2014 compared with the survey in 2011. The proportion of children being fully immunized at 12 months of age increased from an average of 68.8% (95% confidence interval, CI: 67.5–70.1) in 2001 to an average of 88.0% (95% CI: 87.0–89.0) in 2011, only to decline to an average of 82.4% (95% CI: 80.7–84.0) in the most recent survey. The decline seen in the fully immunized coverage in 2014 can be attributed to reductions in polio and DPT coverage.

**Table 2:** Immunization coverage in Nepal, 2001–2014

Vaccine	2001 (DHS)			2006 (DHS)			2011 (DHS)			2014 (MICS)	
	No. of children immunized (no. children eligible for immunization)	Coverage, (%) (95% CI)		No. of children immunized (no. children eligible for immunization)	Coverage, (%) (95% CI)		No. of children immunized (no. children eligible for immunization)	Coverage, (%) (95% CI)		No. of children immunized (no. children eligible for immunization)	Coverage, (%) (95% CI)
BCG (all children surviving first day of life)	5300 (6471)	81.9 (80.9–82.8)		4756 (5252)	90.6 (89.7–91.3)		4705 (5054)	93.3 (92.6–94.0)		2832 (3070)	92.2 (91.2–93.2)
First dose of oral polio (children older than 2 months)	6100 (6211)	98.2 (97.9–98.5)		4902 (5058)	96.9 (96.4–97.4)		4647 (4909)	94.7 (94.0–95.3)		2790 (2939)	94.9 (94.1–95.7)
Second dose of oral polio (children older than 3 months)	5920 (6090)	97.2 (96.8–97.6)		4643 (4942)	93.9 (93.2–94.6)		4475 (4804)	93.2 (92.4–93.8)		2635 (2833)	93.0 (92.0–93.9)
Third dose of oral polio (children older than 4 months)	5548 (5945)	93.3 (92.7–93.9)		4413 (4846)	91.1 (90.2–91.9)		4296 (4708)	91.3 (90.4–92.0)		2424 (2752)	88.1 (86.8–89.3)
First dose of DPT (children older than 2 months)	5126 (6210)	82.6 (81.6–83.5)		4625 (5058)	91.4 (90.6–92.1)		4634 (4909)	94.4 (93.7–95.0)		2736 (2928)	93.4 (92.5–94.3)
Second dose of DPT (children older than 3 months)	4709 (6094)	77.3 (76.2–78.3)		4407 (4974)	88.6 (87.7–89.5)		4460 (4804)	92.8 (92.1–93.6)		2563 (2827)	90.7 (89.5–91.7)
Third dose of DPT (children older than 4 months)	4276 (5949)	71.9 (70.7–73.0)		4194 (4878)	86.0 (85.0–86.9)		4272 (4708)	90.7 (89.9–91.6)		2314 (2744)	84.3 (82.9–85.7)
Measles (children older than 9 months)	3942 (5379)	73.3 (72.1–74.5)		3775 (4421)	85.4 (84.3–86.4)		3788 (4257)	89.0 (88.0–89.9)		2053 (2305)	89.1 (87.7–90.3)
Fully immunized after 12 months (children older than 12 months)	3547 (5154)	68.8 (67.5–70.1)		3460 (4232)	81.8 (80.6–82.9)		3551 (4035)	88.0 (87.0–89.0)		1718 (2084)	82.4 (80.7–84.0)

BCG: bacille Calmette–Guérin; CI: confidence interval; DHS: Demographic and Health Survey; DPT; diphtheria–pertussis–tetanus ; MICS: Multiple Indicator Cluster Survey.

Note: Data from children younger than 5 years were included in the study.

The equity in immunization coverage between wealth quintiles also improved over the study period (Table 3). For children fully immunized at the age of 12 months, the absolute difference in coverage between the wealthiest quintile and the poorest quintile significantly decreased between 2001 and 2014, from an SII of 0.070 (95% CI: 0.061–0.078) to an SII of 0.026 (95% CI: 0.013–0.039). Improvements in equity were also seen for coverage in BCG vaccination, from a SII of 0.054 (95% CI: 0.047–0.061) to anSII of 0.012 (95% CI: 0.005–0.019); in DPT vaccination from 0.068 (95% CI: 0.060–0.076) to 0.016 (95% CI: 0.005–0.026); and in measles vaccination from 0.054 (95% CI: 0.046–0.063) to 0.019 (95% CI: 0.009–0.029). Likewise, the RII improved for these vaccines (Table 3). The equity for polio vaccination coverage did not improve over the study period. However, the SII and RII were already low in 2001: 0.017 (95% CI: 0.012–0.022) and 1.02, respectively, and were similar in 2014 (SII: 0.017; 95% CI: 0.008–0.027 and RII: 1.02). The decrease in polio vaccination coverage during the study period did not seem to affect equity, since the decrease was similar in all wealth quintiles.

**Table 3:** Inequalities in immunization coverage by wealth quintiles, Nepal 2001 and 2014

Vaccination, by wealth quintiles	2001				2014		
	Coverage, (%)	SII (95% CI)	RII		Coverage, (%)	SII (95% CI)	RII
**Fully immunized^a^**							
Richest	84.8	0.070 (0.061–0.078)	1.13		86.0	0.026 (0.013–0.039)	1.03
Fourth	76.6				85.6		
Middle	66.4				83.0		
Second	65.9				83.6		
Poorest	58.6				77.9		
**BCG**							
Richest	92.2	0.054 (0.047–0.061)	1.08		94.4	0.012 (0.005–0.019)	1.02
Fourth	87.2				95.5		
Middle	82.6				95.2		
Second	79.3				94.4		
Poorest	73.9				91.2		
**Three doses of oral polio**							
Richest	97.5	0.017 (0.012–0.022)	1.02		92.8	0.017 (0.008–0.027)	1.02
Fourth	93.6				91.2		
Middle	94.3				87.7		
Second	92.2				87.7		
Poorest	89.6				85.6		
**Three doses of DPT**							
Richest	88.4	0.068 (0.060–0.076)	1.11		89.3	0.016 (0.005–0.026)	1.02
Fourth	78.8				86.7		
Middle	68.7				83.0		
Second	68.7				84.5		
Poorest	62.8				82.0		
**Measles**							
Richest	73.9	0.054 (0.046–0.063)	1.09		93.2	0.019 (0.009–0.029)	1.02
Fourth	67.0				91.1		
Middle	59.3				90.9		
Second	60.0				89.4		
Poorest	54.3				85.9		

BCG: bacille Calmette–Guérin; CI: confidence interval; DPT; diphtheria–pertussis–tetanus; RII: relative index of inequality; SII: slope index of inequality.

^a^ Children who had received BCG vaccine, three doses of diphtheria–tetanus–pertussis vaccine, three doses of polio vaccine and measles vaccines were categorized as fully immunized.

Notes: SII is the absolute difference of immunization coverage between the richest and the poorest wealth quintile.^22^ RII is the ratio of the immunization coverage estimate between wealth quintiles, calculated on the basis of the systematic association between coverage estimate and the socioeconomic status for all wealth quintiles.

**Fig. 1:**
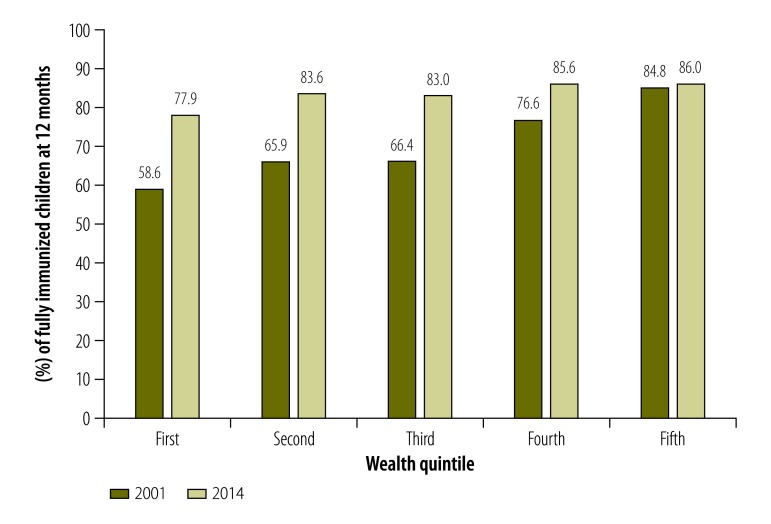
Children fully immunized at 12 months of age, by wealth quintiles, Nepal, 2001 and 2014

Notes: Fully immunized children is a composite variable for children who had received all the scheduled vaccines (bacille Calmette–Guérin, three doses of polio at 1, 2 and 3 months of age, three doses of pentavalent at 1, 2 and 3 months of age and measles at 9 months). The first wealth quintile represents the poorest and the fifth quintile the wealthiest.

For all wealth quintiles, the proportion of children who had received all doses of immunization at the age of 12 months increased during the study period. However, the improvement in coverage was much greater in the poorest quintiles (Fig. 1). For the poorest quintile, coverages of BCG, DPT and measles immunizations improved over time (Table 4). Only coverage of complete polio immunization in the poorest quintiles displays a different pattern with only a minor increase between 2001 and 2006, and then a stagnant situation. The decreased inequity by wealth in immunization coverage was maintained when adjusting for other socioeconomic factors. In a multivariate regression model, we detected a risk reduction for the poorest children (1st quintile) not being fully immunized between 2001 and 2014. In 2001, the poorest children were 1.5-fold less likely not to be fully immunized (aOR: 1.56; 95% CI: 95% 1.37–1.79) compared to children from the other quintiles. In 2014, the likelihood of not being fully immunized was not significant (aOR: 1.17; 95% CI: 0.93–1.46; Table 5). The analysis also revealed a similar trend for mother’s education level. Children of non-educated mothers were four times more likely not to be fully immunized in 2001 (aOR: 4.13; 95% CI: 3.52–4.85), which was reduced to 1.5-fold in 2014 (aOR: 1.53; 95% CI: 1.20–1.96; Table 5).

**Table 4:** Proportion of children in the poorest quintile not vaccinated, Nepal, 2001–2014

Year	Full immunization (at 12 months of age)			BCG (at birth)			DTP3 (at 4 months of age)			Oral polio third dose (at 4 months of age)			Measles (at 10 months of age)	
	No. of nonimmunized children (eligible children)	Proportion, (%) (95% CI)		No. of nonimmunized children (eligible children)	Proportion, (%) (95% CI)		No. of nonimmunized children (eligible children)	Proportion, (%) (95% CI)		No. of nonimmunized children (eligible children)	Proportion, (%) (95% CI)		No. of immunized children (eligible children)	Proportion, (%) (95% CI)
2001	556 (1342)	41.4 (38.8–44.1)		425 (1632)	26.0 (23.9–28.2)		562 (1510)	37.2 (34.8–39.7)		158 (1516)	10.4 (8.9–12.1)		483 (1 388)	34.8 (32.3–37.4)
2006	322 (1075)	30.0 (27.2–32.8)		253 (1328)	19.1 (17.0–21.3)		343 (1233)	27.8 (25.3–30.4)		230 (1233)	18.7 (16.5–20.9)		285 (1 121)	25.4 (20.9–28.1)
2011	228 (1273)	17.9 (15.8–20.1)		204 (1578)	12.9 (11.3–14.7)		236 (1467)	16.1 (14.2–18.1)		228 (1467)	15.5 (13.7–17.5)		223 (1 339)	16.7 (14.7–18.8)
2014	165 (747)	22.1 (19.1–25.2)		96 (1097)	8.8 (7.1–10.6)		177 (984)	18.0 (15.6–20.5)		142 (988)	14.4 (12.2–16.7)		119 (841)	14.1 (11.9–16.7)

BCG: bacille Calmette–Guérin; CI: confidence interval; DPT3; third dose of diphtheria–pertussis–tetanus.

**Table 5:** Likelihood of children aged 12 months not being fully immunized, Nepal, 2001–2014

Variable	aOR (95% CI)			
	2001	2006	2011	2014
**Wealth**				
Nonpoor^a^	Ref.	Ref.	Ref.	Ref.
Poor^b^	1.56 (1.37–1.79)	2.05 (1.71–2.46)	1.70 (1.44–2.22)	1.17 (0.93–1.46)
**Education**				
Literate	Ref.	Ref.	Ref.	Ref.
Illiterate	4.13 (3.52–4.85)	3.12 (2.60–3.75)	3.20 (2.57–4.00)	1.53 (1.20–1.96)
**Residence**				
Hill or terrain	Ref.	Ref.	Ref.	Ref.
Mountain	1.03 (0.82–1.30)	1.55 (1.20–2.01)	0.75 (0.57–0.98)	1.47 (1.14–1.90)
Urban	Ref.	Ref.	Ref.	Ref.
Rural	1.13 (0.85–1.51)	0.63 (0.48–0.82)	0.89 (0.68–1.17)	1.41 (0.96–2.08)
**Sex**				
Boys	Ref.	Ref.	Ref.	Ref.
Girls	1.01 (0.87–1.12)	1.02 (0.99–1.26)	1.12 (0.89–1.27)	1.07 (0.92–1.23)

aOR: adjusted odds ratio; CI: confidence interval; Ref.: reference group.

^a^ Including children in the 2nd to 5th wealth quintiles.

^b^ Including children in the first wealth quintile.

Notes: We adjusted the multivariate logistic regression for sex of child. Children who had received bacille Calmette–Guérin vaccine, three doses of diphtheria–tetanus–pertussis vaccine, three doses of polio vaccine and measles vaccines were categorized as fully immunized.

## Discussion

This study shows that immunization coverage of Nepalese children aged 12 months or younger has improved significantly between 2001 and 2014; an increase that has been accompanied by improved equity. The improved coverage can be attributed to the concentrated efforts of the Nepalese government in collaboration with nongovernmental organizations, which have focused on hard-to-reach and disadvantaged populations with low immunization coverage. Improvements have been the greatest in the poorer and less educated populations, resulting in a decreased equity gap. This achievement is important since the National Immunization Programme strives to achieve herd immunity within a population.^16^ To accomplish such immunity, wide population coverage is needed and failure to reach certain populations might leave them susceptible to vaccine-preventable disease outbreaks.

The results show a setback in polio vaccination coverage. The coverage of the first dose of oral polio vaccine decreased over the study period, from a high level in 2001, to significantly lower between 2001 and 2014. This decrease might be because people may not perceive polio as such a great threat anymore and therefore think immunization is no longer necessary. In 1996, the government initiated a polio eradication programme, which conducted national immunization days targeting all children younger than 5 years with two doses of oral polio vaccine. In subsequent years, Nepal implemented all four strategies required for polio eradication: strengthening routine immunization, supplementary immunization activities, surveillance and outbreak response. The last person infected with indigenous wild polio virus was detected in 1999 and Nepal was declared to have eradicated polio in 2000.^15^ Between 2000 and 2010, 30 individuals in the country have been detected with imported wild polio virus and in the past 5 years, Nepal has been free of polio.

Previous studies conducted in the WHO South-East Asian Region, have examined the relationship between various equity parameters and immunization coverage. In line with our results, a study from Cambodia showed that socioeconomic factors were most strongly associated with decreased access to immunization services, compared to other factors such as gender or rural/urban location.^23^ Furthermore, social distance, that is the distance a person is from health care in terms of acceptability and affordability, was discussed as the main determinant of immunization access as compared to geographical distance.^23^ Our study confirms that geographical location, or the urban/rural divide, does not contribute to differences in immunization coverage. Another study from Pakistan found similar inequities when examining the uptake of measles vaccination.^24^ Children who lived further than 5 km away from vaccination facilities, had poor roofing, parents with a poorly paid job or mothers who were uneducated, were less likely to be vaccinated in both urban and rural areas.^24^ Finally, a systematic review of studies done in India found an association between immunization coverage and the sex of the child, birth order, residence, household wealth, parental education and access to health-care services.^25^ In our study, the sex of the child did not significantly influence coverage. Girls were only slightly less likely to be fully immunized at the age of 12 months compared to boys, a difference that did not show significance at any time point after adjustment for other socioeconomic determinants.

This study has several limitations. First, we used data from two different surveys with different survey methods, which could limit the comparison between time periods. For example, DHS and MICS define and calculate wealth index differently. Second, the absence of ethnicity data in MICS meant that we could not study ethnicity as a social determinant of inequity for immunization coverage. Third, the MICS sample size was smaller than in three previous DHS. Fourth, to calculate SII and RII, the methods require a somewhat even distribution of inequity in the groups compared, otherwise differences in the poorest or richest quintiles compared to the overall population can be hidden and the results misleading.^22^ For our data, there was a large inequity between poorest and richest quintiles, which masked the magnitude of those inequities when calculating the SII. However, using a logistic regression model, we were able to detect these inequities. Fifth, respondent bias can never be ruled out. Respondents might have a willingness to please, which would result in overreporting of immunization coverage. However, this bias can be considered systematic and would not affect the improvement of equity in relation to coverage. Despite these limitations, the survey data allow for comparisons over time, especially since the definition of dependent variables, if the child received a certain vaccine or not, is easy to define, measure and compare.

This study from Nepal demonstrates how directed interventions for the most disadvantaged people can be an important strategy to reach universal immunization coverage and also to help reach Target 3.8 of the sustainable development goals.^26^ Part of this target states that all people should have the right to vaccination, which is also one of the aims of the Human Right to Health Movement.^26^^,^^27^ Even though the increase in overall immunization coverage addressed the equity gap, inequities still exist in Nepal. However, the trend in the past 15 years is promising and indicates the possibility that Nepal will be able to achieve universal immunization coverage.
